# Is It the Surgeon, the Patient, or the Device? A Comprehensive Clinical and Radiological Evaluation of Factors Influencing Patient Satisfaction in 648 Total Knee Arthroplasties

**DOI:** 10.3390/jcm10122599

**Published:** 2021-06-12

**Authors:** Lorenzo Rissolio, Luigi Sabatini, Salvatore Risitano, Alessandro Bistolfi, Umberto Galluzzo, Alessandro Massè, Pier Francesco Indelli

**Affiliations:** 1 Department of Orthopaedic Surgery, “Ospedale Maggiore” of Chieri, 10023 Turin, Italy; rissolio.lor@gmail.com (L.R.); srisitano@gmail.com (S.R.); 2 Department of Orthopaedic Surgery and Traumatology, University of Turin, “Città Della Salute e Della Scienza”-CTO Hospital of Turin, 10126 Turin, Italy; luigisabatini.ort@gmail.com (L.S.); a.bistolfi@libero.it (A.B.); umbertogalluzzo@libero.it (U.G.); alessandro.masse@unito.it (A.M.); 3 Department of Orthopaedic Surgery and Bioengineering, Stanford University School of Medicine and the Palo Alto Veterans Affairs Health Care System(PAVAHCS), Palo Alto, CA 94304, USA

**Keywords:** knee arthroplasty, osteoarthritis, patient satisfaction

## Abstract

Total knee arthroplasty (TKA) is a successful and safe surgical procedure for treating osteoarthritic knees, but despite the overall good results, some patients remain dissatisfied. The aim of this study is to evaluate the influence of patient-related and surgery-related variables in a consecutive group of patients that underwent TKA. Individuals (*n* = 648) who had TKA performed between 01 January 2013 and 31 December 2017 were enrolled in the study. Postoperative Western Ontario and McMaster Universities Osteoarthritis Index (WOMAC) score, Knee Injury and Osteoarthritis Outcome Score (KOOS) and Forgotten Joint score (FJS-12) were collected at a mean follow-up of 4.79 years. Patient satisfaction was assessed with a questionnaire. Determinants of satisfaction (age, sex, smoking, presence of diabetes or cardiovascular disease, pain in other joints, preoperative arthritic stage) and components of satisfaction (slope variation, mechanical axis variation, outlier final alignment, surgeon experience) were examined to identify which variables correlated with positive outcome. Correlations with septic and mechanicals failures were also evaluated. Thirteen percent of patients were unsatisfied, despite good results in KOOS, WOMAC and FJS-12 tests. Female gender, low Kellgren–Lawrence grade and the presence of back pain and pain in other joints were factors associated with poor clinical results. Poorer clinical results were also reported in younger patients. Infection rate was correlated with active smoking and mechanical failure with an outlier final alignment. Comorbidities, smoking habits and high expectations have a big influence on TKA results and on final satisfaction after surgery.

## 1. Introduction

Total knee arthroplasty (TKA) is a successful and safe surgical procedure for treating knees that were severely damaged by arthritis or injuries. The primary indication for TKA is an advanced and painful osteoarthritis affecting the knee function, making it hard for the patient even to perform simple activities of daily living (ADLs), such as climbing stairs or prolonged walking [1].

The incidence of patients undergoing a total knee replacement is growing rapidly: Kurtz et al. [2] projected an increase in the number of TKAs of 143% by 2030, with an expected incidence of 725/100,000. Interestingly, the demand for primary TKA is expected to increase the most in young patients aged from 45 to 55 years old, and the number of TKAs implanted in patients less than 65 could exceed 55% of total procedures [2].

Several advancements have been made in primary TKA that could potentially improve clinical outcomes: computer navigation and robotic surgery, tibial trials with load-sensing devices [3], patient-specific cutting blocks and third-generation TKA designs that have an anatomical femoral component and asymmetrical tibial baseplates. However, numerous studies using more common TKA designs suggest that only from 82% to 89% of TKAs patients are satisfied [4].

The correlation between satisfaction after TKA and ability to perform ADLs is clearly demonstrated in the literature: Nakahara et al. [1] reported that squatting, getting into and out of a car and climbing up or down a flight of stairs, are all activities that significantly correlate with patient satisfaction after TKA. Unfortunately, kneeling and squatting are often limited by having a TKA [5], representing an important reason for dissatisfaction, especially in younger individuals [6].

Two main categories of factors have traditionally affected patient satisfaction: meeting patient expectations and reproducing the normal knee proprioception. If meeting patient expectations is related to a clear patient–surgeon preoperative interaction, then, by considering multiple variables like medical comorbidities and the severity of arthropathy [7], the reproduction of a normal joint proprioception could be influenced by several technical aspects of the procedure, including surgical technique and implant design [8].

In the literature, the relationship between satisfaction and patient-related factors (socio-demographic, medical and psychological) is still debated and not clear. Furthermore, whether final patient satisfaction is influenced by surgical technique and the technology (e.g., implant design) applied during the surgery has not been clearly reported [9]. The classic question “Why TKAs fail in 2020: patient, surgeon or device?” has not been answered, and each stakeholder has their own opinion.

The aim of this study was to evaluate the influence of patient-related factors on postoperative patient satisfaction and final clinical outcome in a consecutive series of primary TKA: two other variables (surgical technique and implant design) remained constant during the completion of the study. The hypothesis of the study was that preoperative patient-related factors played a major role in patients’ reported outcome measures (PROM) and the final outcome, and that preoperative patient optimization is fundamental in order to increase the number of patients that are fully satisfied after arthroplasty.

## 2. Materials and Methods

### 2.1. Inclusion and Exclusion Criteria

A consecutive series of patients with primary and advanced knee osteoarthritis (OA) was enrolled in the study. Patients were treated at Department of Orthopaedic Surgery and Traumatology of CTO Hospital of Turin between 01 January 2013 and 31 December 2017 using the same posterior-stabilized (PS) TKA design (NexGen LPS-Flex, Zimmer-Biomet, Warsaw IN USA). Primary osteoarthritis of the knee was the diagnosis in all cases; exclusion criteria included any preoperative diagnosis of inflammatory or post-traumatic OA of the knee, a body mass index (BMI) greater than 45, previous osteotomies around the knee and the use of constrained condylar and rotating hinged implants [10]. All cases that could not be comprehensively radiologically evaluated by analysing the digital database of the hospital were excluded. The last x-ray control was no older than 12 months. The original study group included 735 TKAs. Eighty-seven patients were lost at the time of final follow-up because they were unreachable (42 patients), deceased (33 patients) or did not consent to the study (12 patients). A total of 648 patients were enrolled in the study. A description of the included population is reported in Table 1.

### 2.2. Operative Technique

The implant used in this study was the NexGen LPS-Flex (Zimmer-Biomet, Warsaw IN, USA), which is characterized by a symmetrical tibial baseplate and a posterior-stabilized femoral component. An identical surgical technique was adopted in all cases: the first surgical step consisted of making a rectangular extension gap (after proximal tibial and distal femur resections, according to the mechanical axis of the knee), which was checked by spacer blocks; at this point, attention was focused on the femur, where the surgical trans-epicondylar (sTEA) axis was drawn and recognized as the main landmark for rotational alignment of the femoral component. After all bone cuts were completed, the PCL was resected in all cases and both gaps were checked one more time, using a spacer block in extension and flexion, and aiming for symmetry between them. An accessory soft tissue release was performed at this point to achieve the desired symmetry between the gaps. After trial component positioning, the trial PS poly insert was used if the surgeon felt that both gaps were well-balanced. The patella was never replaced in this series.

A careful clinical examination was performed under anesthesia with the trial component in place before proceeding to the final implant cementation and standard wound closure. Different surgeons performed the original surgical procedure, and this aspect was considered while analyzing the impact of the surgeon’s experience on the results. We considered any surgeon performing at least 25 implants each year as an “expert” [11]: 343 TKA (53%) were performed by four expert surgeons, while 305 TKA (47%) were performed by another 12 surgeons with a lower volume of knee prostheses per year.

### 2.3. Outcome Measures

#### 2.3.1. Clinical Outcomes

Between February and April 2020, all patients were contacted by phone: the Western Ontario and McMaster Universities Osteoarthritis Index (WOMAC) score [12], Knee Injury and Osteoarthritis Outcome Score (KOOS) [13] and Forgotten Joint Score (FJS-12) [14] were used as validated, patient-reported outcome measures (PROMs). The Forgotten Joint Score (FJS-12) was used to evaluate the extent to which TKAs impacted the everyday lives of patients, and whether the knee joint implant could be forgotten by the patient during normal activities. The results of each score were classified into four categories: EXCELLENT, GOOD, FAIR and POOR as reported in Table 3. Each patient with a bilateral surgery answered different questionnaires for their right and left knees. Moreover, every patient answered YES or NOT to a single, direct question: “in relation to the clinical conditions, pain and daily activities possible for you before the surgery and today, are you satisfied with the surgery?” The decision to use Telemedicine to determine the outcome of the surgical procedure was made as the current study was conducted during the COVID-19 pandemic. The use of digital health programs to monitor patients was reinforced by the administration at the study’s institution, in accordance with the international guidelines [15].

#### 2.3.2. Clinical Exposures

Following the principles addressed by the declaration of Helsinki, a comprehensive evaluation of preoperative patient-related factors (socio-demographic, medical and psychological) was conducted through a chart. Particular attention was paid to the patient’s lifestyle (smoking, drug addiction, alcoholism). Comorbidities prior to surgery were reviewed on the hospital database, recording the presence of heart diseases, diabetes, and pain in other joints, or a back pain. The distribution of these exposures is reported in Table 2.

#### 2.3.3. Radiological Evaluation

All patients followed an identical radiological assessment: anterior–posterior and lateral weight-bearing radiographs, a long-leg view of the lower limbs, and a Merchant view [16] of the patellofemoral joint. These assessments were performed in all patients, preoperatively and at six months, one year and every two years. All radiographs were reviewed by an orthopaedic surgeon who was not part of the surgical team and blinded to the clinical outcome results: the reviewing surgeon evaluated the pre-operative arthritic stage following the Kellgren–Lawrence (K-L) scale [17] and assessed post-operative results according to the Knee Society Roentgenographic evaluation system (KSS) for radiolucency, femorotibial alignment, and evidence of loosening, wear, and osteolysis [13].

Femoral mechanical axis, tibial mechanical axis, hip–knee–ankle angle, mechanical axis of the lower limb and anatomical axis were measured with the Paley method [18] before surgery, and post-operatively in all cases. From the sagittal perspective, the tibial slope was calculated using the posterior tibial cortex as a reference [19]. The degrees of variation after surgery in the mechanical axis (D mechanical axis) and slope (D slope) were kept under consideration to assess if greater changes could influence the clinical results and patient’s satisfaction. Patients with a post-operative mechanical axis, with a discordance of +/−3° from the mean range, were considered outliers.

**Figure 1:**
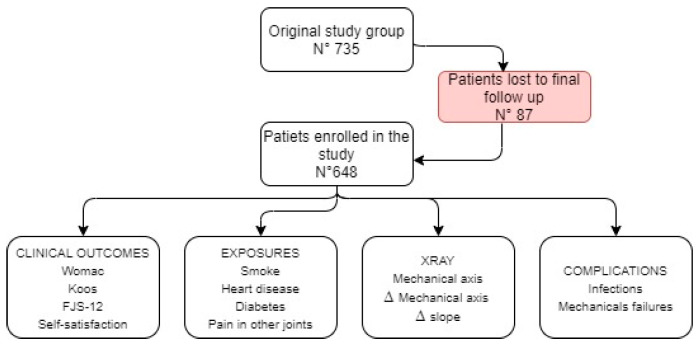
Flow chart describing study demographics and methodology.

The review of the hospital database, the phone interview and the follow-up x-rays were used to identify the rate of infection and mechanical failures that occurred. A flow chart of the materials and methods used is provided in Figure 1.

#### 2.3.4. Statistical Analysis

All collected data were entered into a Microsoft Excel^®^ (Microsoft Corporation, Redmond, WA, USA) file. These data were used to calculate descriptive statistics (average numbers and standard deviations). The statistical analysis was performed through the MedCalc^®^ software (MedCalc Software Ltd., Ostend, Belgium); independent variables were differentiated in terms of the determinants of satisfaction (age, sex, smoke, presence of diabetes or cardiologic diseases, pain in other joints) and components of satisfaction (slope variation, mechanical axis variation, outlier final alignment, surgeon experience). Each variable was examined, with every single questionnaire considered as a dependent variable using logistic regression and considering a *p*-value < 0.05 as statistically significant. Correlations were researched regarding septic and mechanical failures with Odd Ratio calculation. Any association was tested using simple logistic regression.

## 3. Results

### 3.1. Patient Reported Outcome Measurements (PROMs)

The average result according to the WOMAC score was 16.85 (SD ± 22.29) at the final FU: 74% (*n* = 481) reported excellent results, 15% (*n* = 98) good, 6% (*n* = 38) fair and 5% (*n* = 31) poor; thus, 89% of patients reported good or excellent results.

The average KOOS score was 20.05 (SD ± 21.01). Seventy-two percent (*n* = 464) were included in the excellent group, 17% (*n* = 113) in the good one, 8% (*n* = 50) in the fair and 3% (*n* = 21) in the poor one, with 89% of patients reporting excellent or good results.

Regarding the Forgotten Joint Score (FJS-12 4% (*n* = 26) of patients had a poor final score, 9% (*n* = 59) had a fair score, 14% (*n* = 89) had a good score and 73% (*n* = 474) had an excellent score. Interestingly, 559 patients (86.27%) declared themselves satisfied with their TKAs; 13.73% of patients were dissatisfied (Table 3).

### 3.2. Clinical Results

The female gender was ultimately predictive of worse results in all tests (WOMAC: *p* < 0.001, KOOS: *p* = 0.003, FJS-12: *p* = 0.005, Satisfaction: *p* = 0.01), as were back pain and pain in other joints (WOMAC: *p* = 0.016, KOOS: *p* = 0.012, FJS-12: *p* = 0.041, Satisfaction: *p* = 0.03).

Age was not a factor that influenced functional results or the FJS, but younger patients seemed to have a lower grade of satisfaction regarding their TKAs (WOMAC: *p* = 0.56, KOOS: *p* = 0.94, FJS-12: *p* = 0.98, Satisfaction: *p* = 0.04).

No correlation was found with smoking (WOMAC: *p* = 0.09, KOOS: *p* = 0.21, FJS-12: *p* = 0.11, Satisfaction: *p* = 0.47), cardiovascular illnesses (WOMAC: *p* = 0.98, KOOS: *p* = 0.86, FJS-12: *p* = 0.34, Satisfaction: *p* = 0.93) or diabetes (WOMAC: *p* = 0.06, KOOS: *p* = 0.18, FJS-12: *p* = 0.51, Satisfaction: *p* = 0.99).

### 3.3. Radiological Results

The final radiological assessment showed an average anatomical axis after surgery of 3.67° in valgus (SD ± 2.83°), versus a pre-operative 0.39° of varus (SD ± 8.95°). The average difference before and after surgery was of 4.09° (SD ± 8.27°). The mechanical axis varied from an average of 6.13° in varus (SD ± 9.15°) to 2.36° in varus (SD ± 3.59°), with an average difference of 4.48° (SD ± 8.07°). The tibial slope changed from an average of 6.48° (SD ± 4.69°) to 3.14° (SD ± 3.26°) after surgery, with an average variation of 3° (SD ± 5°). The preoperative K-L grade was 3.49, with an SD of ± 0.69.

Regarding the mechanical axis outliers, no statistically significant differences were found in this population, comparing PROMs (WOMAC: *p* = 0.61, KOOS: *p* = 0.58, FJS-12: *p* = 0.82).

Furthermore, no significant correlation was found when comparing clinical and functional results, with differences in the mechanical axis (WOMAC: *p* = 0.62, KOOS: *p* = 0.82, FJS-12: *p* = 0.6) and a different tibial slope (WOMAC: *p* = 0.99, KOOS: *p* = 0.84, FJS-12: *p* = 0.45). A statistically significant correlation was found with pre-operative grade of osteoarthritis. High K-L values were paired with better functionality, comfort and satisfaction results. (WOMAC: *p* = 0.001, KOOS: *p* = 0.001, FJS-12: *p* = 0.008, Satisfaction: *p* = 0.005).

### 3.4. Complications

Implant failure leading to revision surgery was reported in 29 cases; in 13 cases (2%) a deep infection was identified and then treated with a two-stage revision technique. We registered 16 mechanical failures (2.5%): 12 aseptic loosening of the components and four patients had persistent anterior knee pain, requiring patellar resurfacing.

The odds ratios (OR) revealed a strong relationship between smoking habit and septic failure of the implant (OR: 6.67, *p* = 0.001). Diabetes was not significantly correlated with implant failure (OR: 2.27, *p* = 0.06).

Mechanical failures were instead firmly related to outlier alignment (OR: 3.01, *p* = 0.003); evidence increased only if aseptic loosening was considered, excluding patellar revisions. (OR = 5.02, *p* = 0.006).

No difference in outcome was found between the group of patients operated on by an expert knee surgeon and those operated on by a knee surgeon with less expertise (WOMAC: *p* = 0.47, KOOS: *p* = 0.35, FJS-12: *p* = 0.27, Satisfaction: *p* = 0.52).

All statistical results are summarised in Table 4.

## 4. Discussion

The results of this study confirmed the authors’ hypothesis that patient-related factors played a major role in the final outcome. Socio-demographic (female sex and younger age) and medical (radiological degree of knee OA, chronic low back pain, polyarthritis, smoking) factors correlated with a worse clinical outcome and inferior satisfaction rate. On the surgeon side, malalignment (more than 3° from the desired mechanical axis of the lower extremity) was correlated with an increased risk of mechanical failure of the implant.

The overall clinical results, expressed by WOMAC and KOOS indices, were similar to those reported in the literature [20,21]. The average scores were 16.85 (WOMAC) and 20.05 (KOOS), with 89% of patients showing good and excellent results. Comfort with knee implants was evaluated through the Forgotten Joint Score: the average result was 79.9, with 87% of patients having excellent and good results. In contrast, 13.73% of patients declared themselves to not be fully satisfied with their implant: this finding is consistent with other reports in the literature [8]. Major complications leading to failure and revision surgery included periprosthetic joint infections (2%) and aseptic loosening (2.5%), no different from the current literature [22].

Our study confirmed worse clinical results and lower satisfaction in female patients.

This is not the first study showing a demographic difference in terms of satisfaction rate and clinical outcome after TKA: differences between men and women, in terms of knee function after TKA, were previously reported. Parsley et al. [23] showed that women who undergo TKA seek treatment at a later stage than men, and subsequently have greater disability at the time of surgery; this study showed differences between sexes in functional scores after TKA, suggesting that earlier treatment in females would enhance postoperative outcome. Other studies showed that women have more residual pain and stiffness than men after TKA [8,24]. Fisher et al., analysing a group of 71 patients with stiffness and pain after TKA at 1 year of follow-up, found that female gender, as well as a higher BMI and previous knee surgeries, were all associated with worse results [25].

In our study, age was not a factor modifying KOOS, WOMAC and FJS-12 results, but younger patients seemed to be less satisfied with their TKAs when compared to older patients. Interestingly, conflicting data are presented in the literature regarding age and its association with dissatisfaction. Matsuda et al., evaluating 500 TKAs, found a correlation between older age and lower satisfaction [26], while Parvizi et al. [27] reported that 30% of young patients had residual symptoms and limitations after modern design TKA. Williams et al. [28] reported lower Oxford Knee Score (OKS) when patients aged <55 years were compared with all other age groups. The current authors suggest that poor results and less satisfaction are related with higher expectations and functional requests, typical of the younger population.

Osteoarthritis (OA) severity is widely recognized as an important factor modifying pain, function, and satisfaction in TKA patients. In our study, patients with worse pre-operative OA reported better symptom relief and an outcome that better met their expectations.

Valdes at al. demonstrated that patients with lower Kellgren–Lawrence OA scores were more likely to experience higher post-surgical pain [29]. Better clinical results at five years after the original surgery were reported by Keurentjes et al. in patients who underwent primary TKA with an advanced preoperative OA [30].

The general health of patients always represents a concern for the orthopaedic surgeon: diabetes [31] and cardiologic illnesses are quite common among the patients that usually need a TKA; they represented 17.6% and 25.3%, respectively, of the population analysed in the current study. Regardless of this, we did not find a correlation between those comorbidities and the final clinical results and satisfaction rate, consistent with other reports in the literature [32,33]. However, a strong correlation between diabetes and deep postoperative infections was confirmed by this and other studies [34]. Diabetes was also recognised as an important risk factor for infection by the International Consensus Meeting on Periprosthetic Joint Infections [35]; a comparable trend was found in our data (OR = 2.92), but this result was not statistically significant (*p* = 0.06 IC: 0.94–9.11).

Active smoking habit was strongly correlated with a higher infection rate in this study: almost 50% of patients who underwent revision surgery after a deep infection were active smokers, showing an OR of 6.67 (*p* = 0.001). Similar results were reported by Duchman et al. in an extensive revision of more than 78,000 cases [36]. The risk of an early TKA revision related to smoking was also reported by Lim e al. [37]; interestingly, those authors were not able to relate this complication to infections, loosening or other specific causes.

The current authors also analysed surgeon-related factors, since the role of the lower limb’s postoperative alignment has been debated in recent years. Recently, the influence of mechanic alignment on the excellent results achieved after TKA has been questioned, and few surgeons have suggested that it is best to reproduce the patient’s specific physiologic alignment while performing a TKA [38]. The current literature considers “outlier”-aligned TKAs to be those with limbs of more than 3° in terms of valgus or varus deformity, with respect to the ideal 0°, during radiological Hip–Knee–Ankle evaluation. In this study, the outlier alignment and its effect on patient satisfaction was analysed without finding any correlation, indicating that aiming for a mechanically aligned TKA might have an inferior effect than previously thought. However, a significant increased risk of mechanical failure leading to revision surgery was found in those patients with a knee alignment outside the desirable ± 3° (OR 3.01; *p* = 0.003); this risk appeared even higher (OR 5.02, *p* = 0.006) when cases requiring patellar revision were excluded. Due to this finding, the current authors recommend avoiding outlier alignment in order to reduce the risk of mechanical failure, even if clinical outcomes appear unaffected [39].

The same surgical technique was used in all cases presented in this study, but different surgeons performed the procedure. We divided the patients into two groups according to the surgeon’s own experience: surgeons performing 25 or more implants a year were considered “experts”. Interestingly, no significant differences in reported clinical outcome, satisfaction, or complications were found, whether the surgery was performed by a “junior’ or an “expert” surgeon. In contrast, Lau et al. [11] found a significant association between low volume surgeons and higher rate of infection, longer procedure time, longer length of stay, higher transfusion rate and inferior patient-reported outcome.

This study has several major limitations. First, this is a retrospective study and the authors were not able to determine the preoperative clinical and functional scores. Second, final data were reported by patients by phone, and a classical physical examination was not performed. This study was performed during the COVID-19 pandemic and the use of Telemedicine to connect with patients was recommended by several guidelines in the first author’s country [40]. Telemedicine has been shown to be a successful way of gathering clinical data from orthopaedic patients, even when it is not possible to have direct contact with patients [41].

Third, the authors recognize that not all the preoperative socio-demographic, medical and psychological factors present in the patient medical records were analysed for their potential influence on the final clinical outcome and satisfaction rate.

## 5. Conclusions

TKA is a reliable and efficient procedure to treat patients with primary osteoarthritis. The current study confirmed good outcomes in most cases, with 89% of results marked as good and excellent according to the WOMAC and KOOS scores; almost 87% of patients were fully satisfied. Unfortunately, a high rate of dissatisfaction (13%) was still reported, confirming data in the literature. Confirming our first hypothesis, the preoperative patient-related factors played a major role in the final outcome: peri-prosthetic joint infections were detected in 2% of patients, showing a significant correlation with active smoking. Mechanical failures were detected in 2.5% of patients, demonstrating a strong relationship with outlier alignment of the lower limb. Female gender, younger age, lower radiologic stage of osteoarthritis, presence of polyarthritis and presence of low back pain were all associated with worse clinical results. The surgeon was not shown to be a factor in determining clinical outcome and patient satisfaction when compared with the surgical technique and prosthesis design used.

In conclusion, detailed and comprehensive informed consent is mandatory in order to correctly instruct the patient and obtain a satisfactory outcome, especially in populations with high functional requests and expectations. Comorbidities and smoking habit should be investigated and corrected prior to surgery. More clinical studies need to be performed to better understand the causes and remedies of patient dissatisfaction after TKA, and to evaluate the results obtained using new technologies and techniques.

## Figures and Tables

**Table 1:** Patients’ general information.

Mean age (years)	72 (±7.4)
Gender (male:female)	176:472
Side (right:left)	324:324
Bilateral implant (n)	41
Follow-up (years)	4.79
Minimum follow-up (months)	24

**Table 2:** Patients’ exposures *n*(%).

Active smoker	61 (9.4%)
Heart disease	164 (25.3%)
Diabetes	114 (17.6%)
Pain in other joints/back pain	414 (63.9%)

**Table 3:** PROMs results.

	EXCELLENT	GOOD	FAIR	POOR
WOMAC	74% (481)	15% (98)	6% (38)	5% (31)
KOOS	72% (464)	17% (113)	8% (50)	3% (21)
FJS-12	73% (474)	14% (89)	9% (59)	4% (26)

WOMAC: score from 96 to 0. Excellent: 0–23, Good: 24–47, Fair: 48–71, Poor: 72–96. KOOS: score from 100 to 0. Excellent: 0–24, Good: 25–49, Fair: 50–74, Poor: 75–100. FJS-12: score from 0 to 100. Excellent: 75–100, Good: 50–74, Fair: 25–49, Poor: 0–24.

**Table 4:** Statistic results.

	FJS12	KOOS	WOMAC	SATISFACTION	Infection	Mechanical Failure
Female gender	**0.005**	**0.003**	**<0.001**	**0.01**	/	/
Age	0.98	0.94	0.56	**0.04**	/	/
Heart illnesses	0.34	0.86	0.98	0.93	OR:1.29 IC: 0.39–4.23 (*p* = 0.6)	OR: 0.41 IC:0.09–1.84 (*p* = 0.25)
Smoke	0.11	0.21	0.09	0.47	OR: 6.67 IC: 2.1–21.3 (***p* = 0.001**)	OR: 0.71 IC: 0.09–5.5 (*p* = 0.74)
Diabetes	0.51	0.18	0.06	0.99	OR: 2.92 IC: 0.94–9.11 (*p* = 0.06)	OR: 0.31 IC: 0.04–2.39 (*p* = 0.26)
Pain in other joints	**0.041**	**0.012**	**0.016**	**0.03**	OR: 1.25 IC: 0.38–4.1 (*p* = 0.71)	OR: 0.56 IC: 0.21–1.5 (*p* = 0.25)
Surgeon experience	0.27	0.35	0.47	0.52	/	/
Kellgren -Lawrence	**0.008**	**0.001**	**0.001**	**0.005**	/	/
D mechanical axis	0.6	0.82	0.62	0.58	/	OR: 3.01 IC: 1.07–8.47 (***p* = 0.003**)
D slope	0.45	0.84	0.99	0.67	/	/
outliers	0.82	0.58	0.61	0.72	/	/

OR: Odds Ratio. D mechanical axis: degrees variation of mechanical axis after surgery. D slope: degrees variation after of slope surgery.

## Data Availability

All underlying data are in the text.
